# Supplementary material for: Composition and Antibacterial Activity of the Essential Oil from Pimenta dioica (L.) Merr. from Guatemala
Source: Medicines (Basel). 2020 Sep 23;7(10):59. doi: 10.3390/medicines7100059 (PMC7597960; doi:10.3390/medicines7100059)
Supplement: Supplementary file 1 [file medicines-07-00059-s001.pdf]

Supplementary Materials: Composition and Antibacterial Activity of the Essential Oil from *Pimenta dioica* (L.) Merr. from Guatemala

Max Samuel Mérida-Reyes, Manuel Alejandro Muñoz-Wug, Bessie Evelyn Oliva-Hernández, Isabel Cristina Gaitán-Fernández, Daniel Luiz Reis Simas, Antonio Jorge Ribeiro da Silva and Juan Francisco Pérez-Sabino

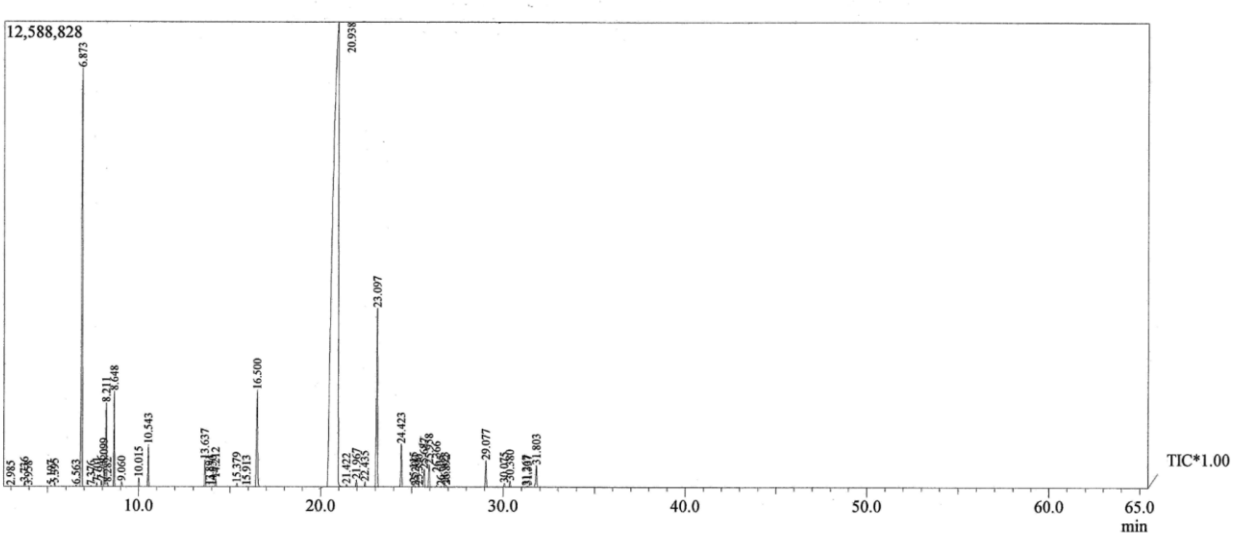

Figure S1. Gas chromatography coupled with mass spectrometry (GC/MS) chromatogram of the essential oil of leaves of *P. dioica*.

Peak Report TIC

Peak#	R.Time	I.Time	F.Time	Area	Area%	Height	Height%	A/H	Mark	Name
1	2.985	2.958	3.008	17317	0.00	11694	0.03	1.48		
2	3.736	3.700	3.792	227424	0.06	113795	0.25	2.00		
3	3.958	3.933	3.983	19733	0.01	13038	0.03	1.51		
4	5.197	5.167	5.233	43621	0.01	23061	0.05	1.89		
5	5.395	5.233	5.433	147707	0.04	74779	0.17	1.98		
6	6.563	6.525	6.600	94031	0.02	44964	0.10	2.09		
7	6.873	6.767	6.925	38326429	10.03	11319023	25.23	3.39		
8	7.376	7.342	7.408	31233	0.01	14840	0.03	2.10		
9	7.701	7.658	7.742	248660	0.07	107765	0.24	2.31		
10	7.946	7.892	8.000	385934	0.10	160926	0.36	2.40		
11	8.099	8.042	8.142	1564516	0.41	622839	1.39	2.51		
12	8.211	8.142	8.258	5958830	1.56	2276075	5.07	2.62	V	
13	8.282	8.258	8.325	216660	0.06	103342	0.23	2.10	V	
14	8.648	8.575	8.708	6681130	1.75	2592059	5.78	2.58		
15	9.060	9.008	9.108	341917	0.09	138183	0.31	2.47		
16	10.015	9.958	10.092	641894	0.17	244328	0.54	2.63		
17	10.543	10.475	10.608	3132999	0.82	1149280	2.56	2.73		
18	13.637	13.567	13.700	2172847	0.57	727401	1.62	2.99		
19	13.891	13.842	13.942	43285	0.01	14931	0.03	2.90		
20	14.050	13.942	14.158	35878	0.01	12474	0.03	2.88		
21	14.212	14.158	14.275	677724	0.18	227050	0.51	2.98		
22	15.379	15.325	15.458	282323	0.07	93788	0.21	3.01		
23	15.913	15.875	15.967	43684	0.01	14433	0.03	3.03		
24	16.500	16.375	16.650	11653126	3.05	2624639	5.85	4.44		

Figure S2. Report of the chromatographic results of GC/MS analysis of the essential oil of leaves of *P. dioica*.

Peak#	R.Time	I.Time	F.Time	Area	Area%	Height	Height%	A/H	Mark	Name
25	20.938	20.342	21.158	272656625	71.37	12579822	28.04	21.67		
26	21.422	21.367	21.475	206758	0.05	71090	0.16	2.91		
27	21.967	21.908	22.025	633514	0.17	208006	0.46	3.05		
28	22.435	22.383	22.500	399009	0.10	137042	0.31	2.91		
29	23.097	22.975	23.167	19776217	5.18	4842099	10.79	4.08		
30	24.423	24.333	24.500	4253518	1.11	1175536	2.62	3.62		
31	25.115	25.050	25.167	355602	0.09	102056	0.23	3.48		
32	25.200	25.167	25.233	53495	0.01	19874	0.04	2.69	V	
33	25.390	25.317	25.442	137577	0.04	41348	0.09	3.33		
34	25.687	25.617	25.767	1728493	0.45	489194	1.09	3.53		
35	25.958	25.767	26.025	2093033	0.55	610196	1.36	3.43	V	
36	26.366	26.317	26.467	159736	0.04	45333	0.10	3.52		
37	26.605	26.467	26.650	61400	0.02	21059	0.05	2.92		
38	26.823	26.650	26.867	362905	0.09	103521	0.23	3.51		
39	26.892	26.867	26.967	133693	0.03	46318	0.10	2.89	V	
40	29.077	28.992	29.150	2536242	0.66	728253	1.62	3.48		
41	30.075	30.017	30.133	329787	0.09	102712	0.23	3.21		
42	30.380	30.308	30.450	557790	0.15	166393	0.37	3.35		
43	31.267	31.225	31.308	57024	0.01	16042	0.04	3.55		
44	31.337	31.308	31.392	87989	0.02	30634	0.07	2.87	V	
45	31.803	31.633	31.875	2488312	0.65	604894	1.35	4.11		
				382057621	100.00	44866129	100.00			

Figure S3. Continuation of report of the chromatographic results of GC/MS analysis of the essential oil of leaves of *P. dioica*.

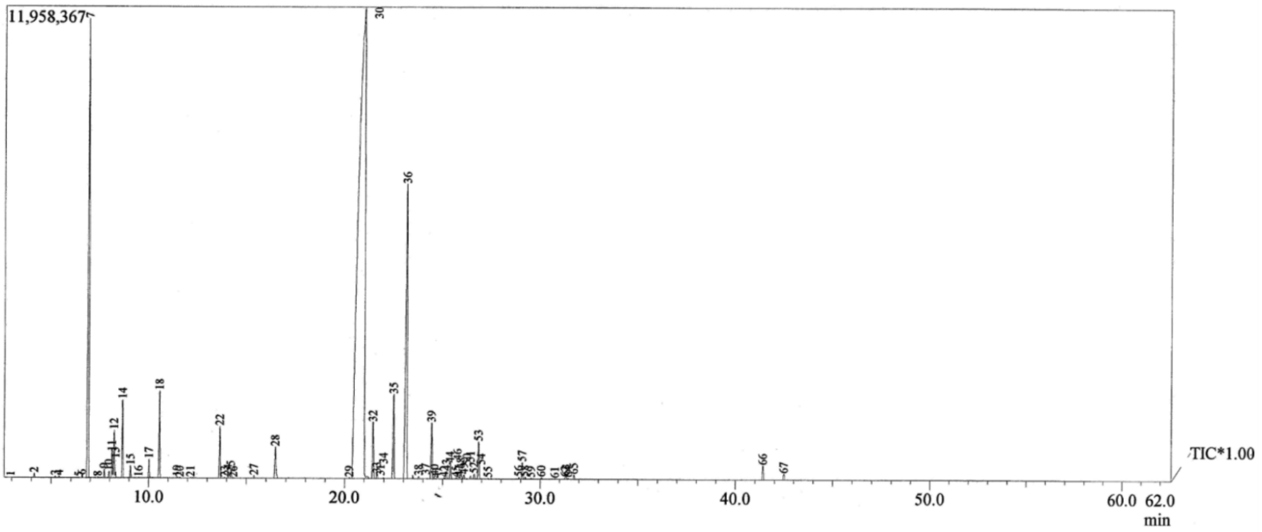

Figure S4. GC/MS chromatogram of the essential oil of leaves of *P. dioica*.

Peak Report TIC

Peak#	R.Time	I.Time	F.Time	Area	Area%	Height	Height%	A/H	Mark	Name
1	2.677	2.650	2.708	17385	0.00	11841	0.02	1.47		
2	4.141	4.100	4.192	199540	0.05	110132	0.22	1.81		
3	5.207	5.167	5.242	76991	0.02	41189	0.08	1.87		
4	5.405	5.367	5.442	93840	0.02	49726	0.10	1.89		
5	6.414	6.383	6.450	24952	0.01	12786	0.03	1.95		
6	6.573	6.450	6.617	137579	0.03	63065	0.12	2.18		
7	6.889	6.775	6.942	42250592	10.11	11641771	23.01	3.63		
8	7.385	7.350	7.425	56647	0.01	25131	0.05	2.25		
9	7.711	7.667	7.758	519786	0.12	219227	0.43	2.37		
10	7.955	7.900	8.008	411343	0.10	171333	0.34	2.40		
11	8.107	8.050	8.150	1678777	0.40	652863	1.29	2.57		
12	8.216	8.150	8.267	3265155	0.78	1209370	2.39	2.70	V	
13	8.290	8.267	8.342	304616	0.07	143899	0.28	2.12	V	
14	8.655	8.583	8.717	5235947	1.25	1986062	3.93	2.64		
15	9.069	9.017	9.125	780580	0.19	310149	0.61	2.52		
16	9.498	9.467	9.533	25480	0.01	12180	0.02	2.09		
17	10.024	9.967	10.117	1333812	0.32	479430	0.95	2.78		
18	10.563	10.475	10.642	6497505	1.55	2207594	4.36	2.94		
19	11.475	11.442	11.517	49585	0.01	24661	0.05	2.01		
20	11.601	11.567	11.633	20129	0.00	9629	0.02	2.09		
21	12.157	12.125	12.192	28106	0.01	15996	0.03	1.76		
22	13.650	13.575	13.717	4030580	0.96	1318339	2.61	3.06		
23	13.895	13.850	13.950	52044	0.01	18004	0.04	2.89		
24	14.064	13.950	14.125	159851	0.04	54399	0.11	2.94		

Figure S5. Report of the chromatographic results of GC/MS analysis of the essential oil of fruits of *P. dioica*.

Peak#	R.Time	I.Time	F.Time	Area	Area%	Height	Height%	A/H	Mark	Name
25	14.217	14.125	14.300	447491	0.11	149760	0.30	2.99		
26	14.337	14.300	14.375	24396	0.01	11727	0.02	2.08		
27	15.383	15.333	15.458	193997	0.05	68431	0.14	2.83		
28	16.472	16.392	16.625	3308345	0.79	806145	1.59	4.10		
29	20.244	20.192	20.292	65222	0.02	23028	0.05	2.83		
30	20.971	20.350	21.075	275548084	65.91	11958367	23.64	23.04		
31	21.149	21.075	21.317	459289	0.11	61003	0.12	7.53	V	
32	21.446	21.317	21.542	5019821	1.20	1438204	2.84	3.49	V	
33	21.646	21.542	21.708	382743	0.09	125813	0.25	3.04	V	
34	21.981	21.942	22.025	69680	0.02	25646	0.05	2.72		
35	22.489	22.383	22.558	7829388	1.87	2146585	4.24	3.65		
36	23.144	22.983	23.208	38172404	9.13	7442147	14.71	5.13		
37	23.471	23.408	23.525	303184	0.07	95258	0.19	3.18		
38	23.777	23.733	23.833	191876	0.05	66180	0.13	2.90		
39	24.437	24.342	24.517	5174729	1.24	1430826	2.83	3.62		
40	24.585	24.517	24.633	222894	0.05	72266	0.14	3.08		
41	24.692	24.633	24.733	43461	0.01	12595	0.02	3.45	V	
42	25.082	25.033	25.133	101560	0.02	34614	0.07	2.93		
43	25.200	25.133	25.267	678219	0.16	199484	0.39	3.40	V	
44	25.403	25.267	25.475	1472204	0.35	401660	0.79	3.67		
45	25.683	25.625	25.717	112511	0.03	35503	0.07	3.17		
46	25.776	25.717	25.825	466412	0.11	132333	0.26	3.52	V	
47	25.858	25.825	25.892	167469	0.04	53495	0.11	3.13	V	
48	25.948	25.892	26.025	895600	0.21	243682	0.48	3.68	V	
49	26.101	26.025	26.183	389193	0.09	112567	0.22	3.46	V	
50	26.258	26.183	26.292	36327	0.01	13110	0.03	2.77		
51	26.434	26.375	26.492	250363	0.06	78619	0.16	3.18		
52	26.618	26.492	26.683	433560	0.10	131453	0.26	3.30		
53	26.839	26.683	26.925	3365141	0.80	950278	1.88	3.54	V	
54	26.983	26.925	27.025	58381	0.01	14266	0.03	4.09	V	
55	27.334	27.283	27.383	69827	0.02	23942	0.05	2.92		
56	28.910	28.833	28.958	127330	0.03	48359	0.10	2.63		
57	29.079	28.958	29.150	1538820	0.37	432101	0.85	3.56		
58	29.228	29.175	29.283	167974	0.04	57163	0.11	2.94		
59	29.536	29.492	29.575	86207	0.02	31975	0.06	2.70		
60	30.075	30.000	30.125	147144	0.04	45610	0.09	3.23		
61	30.765	30.708	30.808	47475	0.01	17321	0.03	2.74		
62	31.283	31.225	31.308	167692	0.04	50537	0.10	3.32		
63	31.344	31.308	31.392	276255	0.07	84121	0.17	3.28	V	
64	31.444	31.392	31.492	98702	0.02	34016	0.07	2.90	V	
65	31.735	31.650	31.825	456154	0.11	115857	0.23	3.94		
66	41.411	41.333	41.483	1257238	0.30	368481	0.73	3.41		
67	42.488	42.417	42.558	512253	0.12	153580	0.30	3.34		
				418087837	100.00	50586884	100.00			

Figure S6. Continuation of report of the chromatographic results of GC/MS analysis of the essential oil of fruits of *P. dioica*.
